# Farmers’ Preferences for Future Agricultural Land Use Under the Consideration of Climate Change

**DOI:** 10.1007/s00267-016-0720-4

**Published:** 2016-07-02

**Authors:** Ulrike Pröbstl-Haider, Nina M. Mostegl, Julia Kelemen-Finan, Wolfgang Haider, Herbert Formayer, Jochen Kantelhardt, Tobias Moser, Martin Kapfer, Ryan Trenholm

**Affiliations:** 1 Institute of Landscape Development, Recreation and Conservation Planning, University of Natural Resources and Life Sciences Vienna, Peter Jordan Straße 82, 1190 Vienna, Austria; 2 School of Resource and Environmental Management (REM), Simon Fraser University, 8888 University Drive, Burnaby, BC V5A 1S6 Canada; 3 Institute of Meteorology, University of Natural Resources and Life Sciences Vienna, Peter Jordan Straße 82, 1190 Vienna, Austria; 4 Institute of Agricultural and Forestry Economics, University of Natural Resources and Life Sciences Vienna, Feistmantelstraße 4, 1180 Vienna, Austria

**Keywords:** Decision-making, Climate change, Environmental premiums, Agri-environmental protection schemes, Land use change, Biodiversity, Austria

## Abstract

Cultural landscapes in Austria are multifunctional through their simultaneous support of productive, habitat, regulatory, social, and economic functions. This study investigates, if changing climatic conditions in Austria will lead to landscape change. Based on the assumption that farmers are the crucial decision makers when it comes to the implementation of agricultural climate change policies, this study analyzes farmers’ decision-making under the consideration of potential future climate change scenarios and risk, varying economic conditions, and different policy regimes through a discrete choice experiment. Results show that if a warming climate will offer new opportunities to increase income, either through expansion of cash crop cultivation or new land use options such as short-term rotation forestry, these opportunities will almost always be seized. Even if high environmental premiums were offered to maintain current cultural landscapes, only 43 % of farmers would prefer the existing grassland cultivation. Therefore, the continuity of characteristic Austrian landscape patterns seems unlikely. In conclusion, despite governmental regulations of and incentives for agriculture, climate change will have significant effects on traditional landscapes. Any opportunities for crop intensification will be embraced, which will ultimately impact ecosystem services, tourism opportunities, and biodiversity.

## Introduction

Austria is characterized by a great diversity of cultural landscapes, which add value to the economy of many regions. These landscapes usually have multiple functions: in addition to agricultural production, they also offer a range of ecosystem services and provide the setting for recreational and touristic landscape experiences. Cultural landscapes and the many factors that influence their quality [e.g., location and policy, such as Agri-environmental schemes (AES)] have already been researched extensively (Primdahl et al. 2003; Kantelhardt 2003; Swetnam et al. 2004; Osterburg et al. 2007; Röder et al. 2006; von Haaren and Bathke 2008; Espinosa-Goded et al. 2010). Particularly, expert-based research has attempted to predict the future development of these landscapes (Hiess 2002) and visualizations were used to illustrate potential consequences for Europe (Aufmkolk 1998; Tahvanainen et al. 2002; Heißenhuber et al. 2004; Kapfer and Ziesel 2008) and other countries (for US see a summary by Bergstrom and Ready 2009, for China see Grosjean and Kontoleon 2009; Jianjun et al. 2013).

As we know from many studies, climate change will likely alter agro-economic use patterns, explicitly in Central Europe (Rogers et al. 2012; de Wit 2006, Kromp-Kolb et al. 2007), which challenges the current standard predictions for future agricultural land use. Two aspects need to be considered: Firstly, agricultural crop production is already changing because the underlying biophysical conditions of the agro-ecosystem resources and functions have already shifted during the past decades due to climate change (Assad et al. 2004; Perarnaud et al. 2005). Secondly, land use has been adapted in order to mitigate climate change, e.g., by the introduction of short-rotation forests (Paulrud and Laitila 2010; Shoyama et al. 2013).

In recent years, several attempts have been made to assess the risk that future climate change effects pose for crop production and to search for suitable adaptation measures for agricultural systems in Central Europe (Eitzinger et al. 2009; Marracchi et al. 2005). The climate change predictions in these models usually assumed future constraints on agricultural production triggered by a limited availability of water and/or increased temperature, while the potential for new types of agricultural production has received less attention (Soja and Pascual-Rodriguez 2010). Furthermore, at least in Austria, predictions on the potential influence of climate change are usually based on qualitative expert opinion only (Eitzinger et al. 2009; Freyer and Dorninger 2010; Kromp-Kolb et al. 2007; Tappeiner et al. 2007; Hiess 2002), assuming that their perception of climate change and experience in decision-making are sufficient to represent the complex business decisions that farmers face. In contrast, Grothmann and Patt (2005) note that research on climate change and land use often ignored farmers’ personal perception of adaptation opportunities and necessity (Grothmann and Patt 2005: 44f). Recent agro-economic research has shown that, in addition to economic indicators (profitability, liquidity, and stability), noneconomic factors such as tradition, education, and social context matter significantly, as documented for AES (Morris 2000; Vaselembrouck et al. 2002; Swetnam et al. 2004; Ruto and Garrod 2009), genetically modified crops (Breustedt et al. 2008), biodiversity conservation (Pagiola et al. 2004; Pröbstl and Zimmermann 2010), and climate change adaptation (Paulrud and Laitila 2010). All of these studies identified significant heterogeneity among farmers. Further research, which investigated links between farmer adaptation to climate change and various elements of social, human, natural, physical, and financial capital, reported a strong influence of education level, experience of past conditions, strong community leadership, social norms regarding environmental action, economic viability of farm businesses, and farm size (de Wit 2006; Hogan et al. 2011; Leith and Haward 2010; Crimp et al. 2008; Milne et al. 2008). In addition, Rogers et al. (2012) highlight the complex nature of rural landholders’ decision-making in the context of climate change. Farmers are the crucial decision makers, who ultimately determine the production systems implemented and the visual appearance of these landscapes and do not necessarily strive to maximize income or economic return when faced with changing baseline conditions. Their decision may not be in line with scientific modeling of climate change and scientific predictions of adaptation processes in land use.

In addition to climate change, economic indicators and noneconomic factors, farmers’ adaptation strategies will also be influenced by agricultural and conservation policy instruments such as AES and other environmental premiums and incentives steering adaptation.

Farmers need to be recognized as crucial decision makers when it comes to the implementation of agricultural climate change policies. Therefore, their decisions may not be in line with expert’s predictions and modeling of adaptation processes in land use, as multiple internal factors may play a major role. This study improves the understanding of farmers’ choices under the consideration of climate change, varying economic conditions, and different policy regimes. In order to discuss the likelihood of land use changes, we selected a study area in Austria mainly characterized by marginal land of high biodiversity and beauty, so changing climatic conditions will allow new land use options for these sites and may require the application of new policy instruments to maintain the current cultural landscape.

The main research objectives are as follows:

- To understand individual farmers’ perception of human-induced climate change, related opportunities, and risks.
- To identify and determine the acceptance of different scenarios of agricultural land use under conditions of climate change.
- To gain insight into farmers’ decision-making processes and, explicitly, into the influence of incentives and structural policies.

## Selected Site and its Conditions Under the Effects of Climate Change

**Fig. 1:**
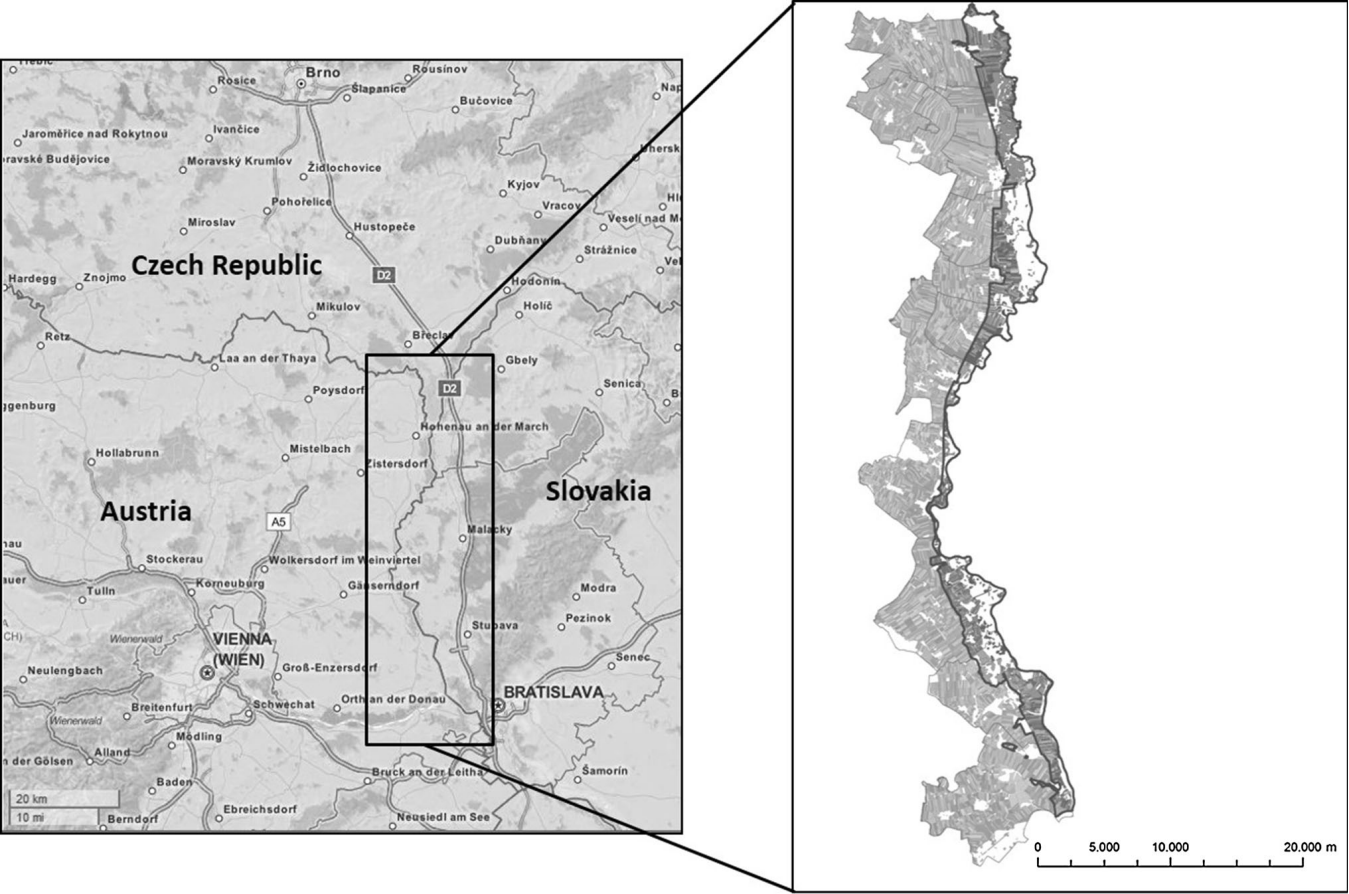
Location of the March–Thaya floodplains, to the Northeast of Vienna [after Open Street Map (*left map*) and Wirth et al. 2013: 25 (*right map*)]

In the past, research on landscape and climate change has largely concentrated on changes with negative effects on agriculture, such as droughts, but has often overlooked potential positive effects accruing from new opportunities. Therefore, this research was undertaken in the March–Thaya floodplains in north-eastern Austria, bordering Slovakia (Fig. 1), where farmers are likely to benefit from climate change. Increasing temperatures and improving growing conditions will lead to new land use options and (most likely) to altered local farmer behavior.

Current meteorological research shows that the global near-surface temperature in Central Europe is expected to follow the past trends of warming. This general warming can be observed by comparing the average near-surface temperature of 1961–1990 with the prognosis for the period of 2021–2050. The overall annual mean temperature in Europe will rise by up to approximately 2 °C by 2050 and 3.5 °C (more than 4.5 °C in the northern regions) by 2100. A temperature increase of up to 4 °C is likely to be expected during the summer months in Southern Europe, as well as the southern parts of Austria by the end of the century (van der Linden and Mitchell 2009). Precipitation predictions for Europe show two clearly separated regions with converse developments. By the end of the century, annual precipitation in Southern Europe and the Iberian Peninsula will gradually decrease. At the same time, precipitation in the northern parts, particularly in Scandinavia and Russia, will significantly increase (van der Linden and Mitchell 2009). Central Europe and the Alps will become a transition region with no significant change in annual precipitation but seasonal shifts.

The climate in the test site is continental with hot, dry summers and cold winters with little snow. During the summer months, low humidity and minor dew formation are typical. The annual mean temperature for the period of 1971–2000 was 9.5 °C (ZAMG 2011). Total annual precipitation averaged 525 mm during the same period, with a slight peak in the summer and a second peak in the fall. Formayer (2007) analyzed the influence of climate change on local temperature. In comparison to the period of 1971–2005, the annual temperature sum will increase from 2383 to 2582 °C in 2050. Moreover, the growing season is likely to be extended from 228 to 245 days, and the dormant season will therefore decrease from 137 to 120 days for the same period. The overall sum of temperatures is likely to increase by about 8 % over the next two decades (Eitzinger et al. 2008).

The temperature increase of 2 °C by 2050 will most likely lead to significant changes of land use in the March–Thaya floodplains. Temperature-induced yield reductions will probably be compensated by the CO_2_ fertilizing effect (Eitzinger 2010). Summer drought will reduce yield stability, especially in the nonirrigated regions of the test site (Eitzinger et al. 2009). The length of the growing season will clearly increase by 12 days (2025 low climate sensitivity scenario) to 32 days (2050 high climate sensitivity scenario) (Eitzinger et al. 2008). By 2050, the growing season for permanent crops will, on average, start 14 days earlier. Yield of winter grain will benefit from this development while summer grain yields will decrease. Also, higher spatial differences in yield will occur in association with site-specific soils’ water storage capacity (Gerersdorfer and Eitzinger 2010; Eitzinger et al. 2008). Overall, yield will in fact increase, yet volatility of these yields will also increase due to more extreme weather conditions. Generally, climate change may not necessarily have a negative impact on agricultural production within the study region (Formayer 2007; Gandorfer and Kersebaum 2009). It is projected that a change of the underlying conditions can easily be compensated through an adjustment of plant cultivation and management strategies, and the already existing or expanded irrigation technology (Wirth et al. 2013).

The test site covers 45,200 ha and 13 small municipalities. About 75 % of the area is intensively used as agricultural land. Here, crop rotation is dominated by winter wheat, spring barley, and sugar beets. Furthermore, the production of corn has increased significantly in recent years. The remaining 25 % of the area is of high nature conservation value (designated as Ramsar and Natura 2000 sites under the EU Habitats and Birds directives), containing cultivated and noncultivated zones. Typical for this part of Austria are relatively large farms with an average size of 60 ha (up to 500 ha). The majority of farms only have a small portion of their land located within the Ramsar area, which has been mainly used as permanent grassland in the past.

This research focuses on these diverse grassland ecosystems with a high relevance for ecosystem services in the region (e.g., biodiversity, soil protection, water retention, recreation) and a well-adapted, extensive land use, significantly supported by AES. Under conditions of climate change, these wetlands will allow farmers to successfully apply alternative land use options and are likely to be changed over time.

## Methodology

### Concept

**Fig. 2:**
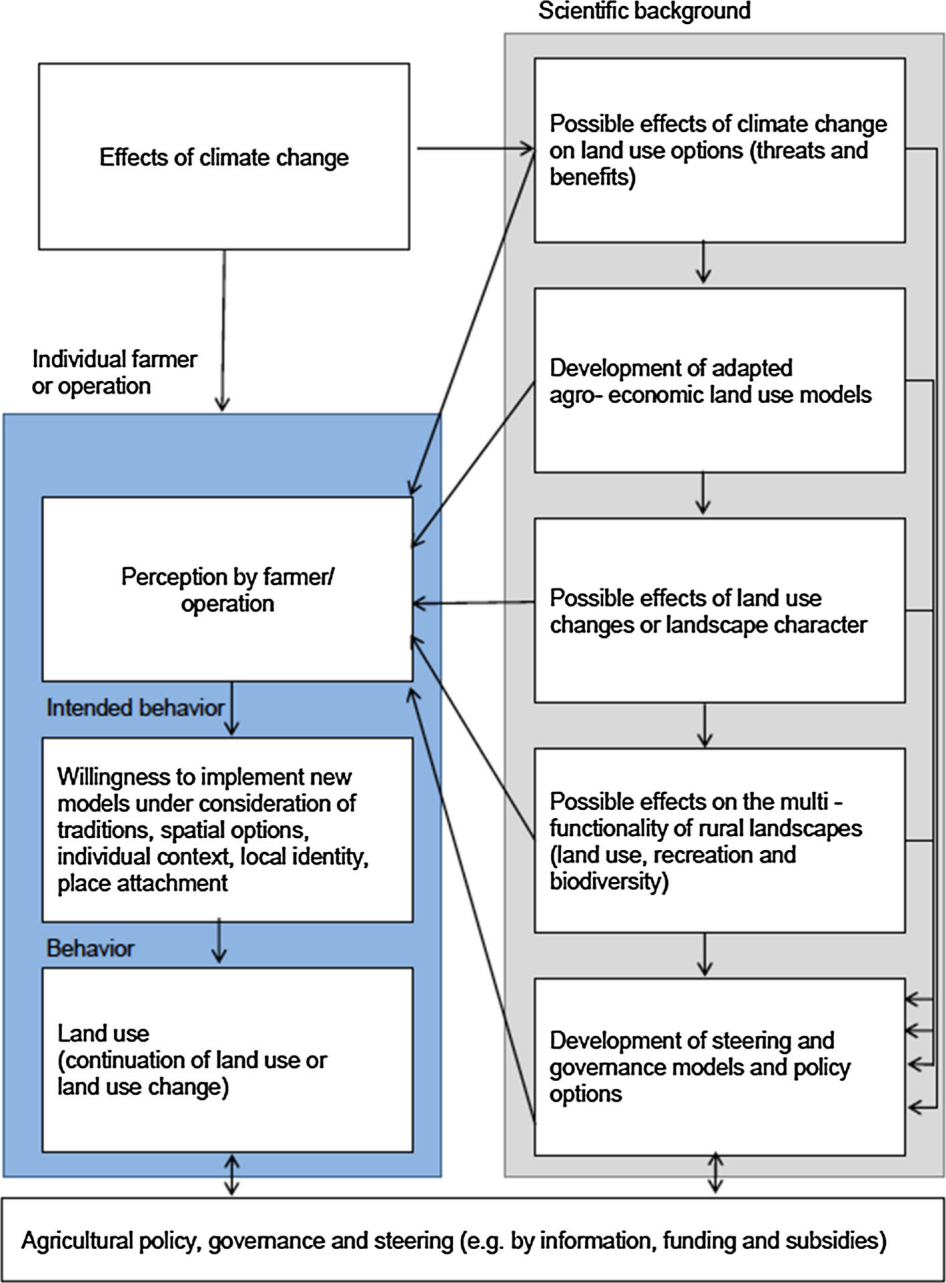
Conceptual framework

As elaborated above and illustrated in Fig. 2, the study first analyzed possible effects of climate change in the selected test site. The second step of the analysis focused on possible agro-economic land use models and possible changes in the contribution margin for both new and traditional land use options. Based on these climate change scenarios and a literature review, various new farming opportunities, such as short-rotation forests, were identified as feasible alternatives. Several stakeholder meetings were arranged to obtain qualitative evaluations from farmers about farming opportunities under a new climate regime, and to test their reaction to environmental premiums or AES, both based on the current programs at the time and the budget plans for the EU funding period of 2014–2020. A typical AES requires farmers to modify farming practices in exchange for a per-hectare payment. The AES are perceived as suitable instruments for steering land use and safeguarding various societal interests. During the last EU funding period, around 6.8 billion € of the EU’s budget were needed to fund these schemes. This research was undertaken during the controversial discussions about new AES concepts for the period 2014–20, and therefore discusses different policy options for steering the overall development in the study region. The steps in light gray in Fig. 2 served as the basis for the farmer questionnaire.

### Questionnaire

In order to investigate perception of farmers and operations, we developed an online questionnaire. This questionnaire was designed as an online questionnaire, but programed to run on laptop computers without requiring access to the Internet. Stakeholders and other nonspecialists pretested the questionnaire to guarantee comprehensibility and ease of operation. Interviews were coordinated in cooperation with the chamber of agriculture and conducted in the March–Thaya floodplains between January and September 2012. The local chamber of farmers, a subassembly of the chamber of agriculture, an institution with mandatory membership for every farmer in Austria, recommended participation in the survey and organized six meetings in the study area where all local farmers were able to fill in the questionnaire. To avoid any sampling bias, farmers who did not participate in the organized meetings were visited on their farms and were asked to complete the survey at home. The cooperation with the chamber increased the response rate significantly. Participating farmers owned approximately 34 % of the entire study area.

#### Questionnaire Set-up

The questionnaire consisted of two sections. The first part included 20 dichotomous, multiple choice, ordinal scale, rating scale, and open-ended questions about farm structure, perception of climate change, planned development of the farm, landscape preferences, and socio demographic questions. For this publication, we particularly focus on two general questions regarding (1) climate change perception and (2) farmer’s perception of future farm development (see sections 4.2 and 4.3 for wording). The second part contained a choice experiment.

#### Choice Experiment Background

The present study positions the choice experiment (CE) to explain farmers’ land use decisions when facing climate change and possible agro-economic adaptation strategies. The CE allows us to analyze different influencing factors on farmers’ decision-making, such as expected market price levels, financial and natural risks, and environmental and agricultural policies (Adamowicz et al. 1998). In addition, this approach permits the incorporation of risk, uncertainties, and hypothetical futures of climate change (van Beukering and Cesar 2004). In other words, we considered the decisions farmers typically face in such a situation as a multivariate decision problem, consisting of a combination of possible policy and outcome alternatives. The choice experiment (CE) allows us to model intended behavior, and recognize that complex decisions are based on several factors considered simultaneously(Hanley et al. 2001; Louviere et al. 2000). CEs are grounded in random utility theory (RUT), which assumes that a decision maker maximizes utility by always choosing the alternative with the highest benefit and that probabilities of choice can be estimated aggregately by following a regression model (McFadden 1974; Ben-Akiva and Lerman 1985; Train 2009). The RUT further presumes, that the total utility consists of a deterministic and a random component. An individual’s *n* utility of choice is therefore described by the function *U*_in_ = *V*_in_ + *ε*_in_, where *U*_in_ is the overall utility of a good (*i*) that is composed of *V*_*in*_ (the deterministic vector of attributes) and *ε*_in_ (the random component of a respondent’s choice). An alternative ***i*** is chosen over alternative *j* if *U*_in_ > *U*_jn_ for all *j* ≠ *i*. Regardless of the assumption that behavior is deterministic on the individual level, all data are modeled aggregately as a total sum of a random process. Hence, the probability of choosing alternative *i* over alternative *j* can be calculated as

1 $${\text{Prob}}(i\left| C \right.) = {\text{Prob}}\left\{ {V_{i} + \varepsilon _{i} {\text{ }}>V_{i} + \varepsilon _{j} ;\quad \forall j \in C} \right\},$$

where *C* refers to the complete set of all possible alternatives. Assuming the error term (*ε*) to be Gumbel-distributed, the probability of choosing alternative *i* can be computed as

2 $${\text{Prob}}(i) = \frac{{\exp \;^{{v_{i} }} }}{{\sum\limits_{j \in C} {\exp \;^{{v_{i} }} } }}$$

which is the standard form of the conditional logit model used in this study (McFadden 1974; Train 2003).

Within the economics literature, CEs have become an established valuation method (Bateman et al. 2002; Hensher et al. 2005) and have lately been applied to the evaluation of ecosystem services (de Groot and Hein 2007). Despite a higher cognitive burden, the multiattribute approach of CEs is still advantageous, as (1) economic values are not elicited directly but are inferred, which decreases strategic response behavior when it comes to payments (Van Beukering and Cesar 2004); (2) the incidence of ethical protesting seems to be lower (Hanley et al. 2001); (3) they excel in measuring nonuse values (Adamowicz et al. 1998); (4) they allow for a deeper understanding of trade-offs between different attributes (Adamowicz et al. 1998), and as (5), in the context of nonmarket ecosystem service valuation, individuals can evaluate nonmarket benefits or hypothetical futures and options in an intuitive and meaningful way (Van Beukering and Cesar 2004). In addition, the choice experiment permits a combined analysis of multiple aspects of hypothetical (future) attributes and scenarios (e.g., impact of funding). Thus, CEs are deemed particularly suitable for the application in climate change research, as currently nonexisting criteria and scenarios (i.e., increased yield through prolonged cultivation periods and new types of funding schemes and subsidies) can easily be integrated alongside existing adaptation strategies into the survey and subsequent analyses. Finally, the method can also accommodate risks and uncertainties into the evaluation and trade-offs.

As Hanley et al. (2001) stated, “CE seems to be ideally suited to inform the choice and design of multidimensional policies.” Therefore, it is not surprising, that the use of CEs to study farmer behavior has increased in the last decade (Scarpa et al. 2003; Birol et al. 2006; Peterson et al. 2007; Roessler et al. 2008; Ruto et al. 2008; Breustedt et al. 2008; Paulrud and Laitila 2010; Asrat et al. 2010; Shoyama et al. 2013). This study belongs to the few studies (Espinosa-Goded et al. 2010; Ruto and Garrod 2009) integrating aspects of the AES into a choice set.

**Fig. 3:**
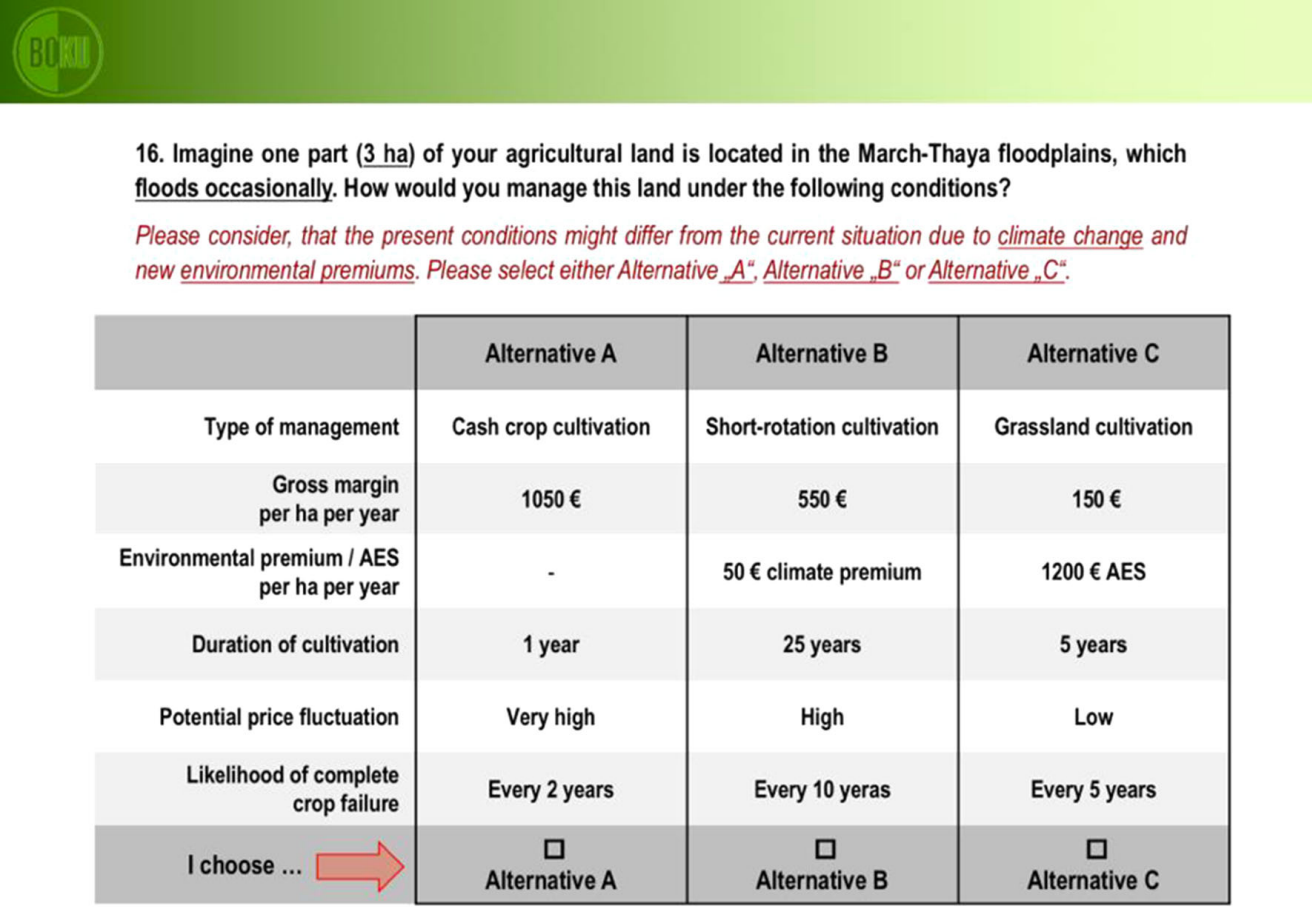
A sample choice set of the choice experiment

The operationalization of the CE required a statistical design plan to create the hypothetical alternatives and their organization into choice sets. The study applies an orthogonal fractional factorial design plan of Resolution III (Raghavarao et al. 2011) in a labeled CE (Fig. 3). This approach ensured that the main effect of each attribute was entirely uncorrelated with all other attributes within the same alternative and every attribute of the other two competing alternatives. The CE required the farmers to choose between three labeled alternatives: cash crop, short-rotation, and grassland cultivation. Each alternative was specified with five attributes (Table 1). The entire design plan contained 48 choice sets, and one respondent evaluated six of these choice sets, which were randomly chosen. For the next respondent, another six randomly selected choice sets without replacement were drawn, until the pool of 48 sets was exhausted; thereafter, another round of choice set application started. Linear by linear interactions were estimable within each alternative separately and integrated in the analyses to explain additional coherences.

**Table 1:** Attributes and levels used in the choice experiment

	Alternative A	Alternative B	Alternative C
Type of management	Cash crop cultivation	Short-rotation cultivation	Grassland cultivation
Gross margin per ha per year	€ 300 € 450 € 750 € 1200 € 1650	€ 150 € 375 € 550 € 725	€ 75 € 150 € 250
Environmental premium per ha per year (AES)	None	None	Austrian AES-funding € 300
	Greening premium € 50	Climate premium € 50 €	Austrian AES-funding € 600
	Greening premium € 150	Climate premium € 100	Austrian AES-funding € 900
		Climate premium € 150	Austrian AES-funding € 1200
Duration	1 year	15 years	7 years
		20 years 25 years	
	[rotation period]	[rotation period]	[contract period]
Potential price fluctuations	Low	Low	Low
	Medium	Medium	
	High	High	
	Very high		
Likelihood of complete crop failure	Every 2 years	Every 10 years	Every 5 years
	Every 3 years	Every 25 years	Every 10 years
			Every 15 years

#### Choice Experiment Attributes and Levels

Each farmer was asked to imagine that one part (3 ha) of his/her agricultural land is located in the March–Thaya floodplains, which floods occasionally. When selecting one of these three labeled alternatives (cash crop, short-rotation forest, grassland cultivation), farmers had to trade-off all attributes associated with each of the alternatives simultaneously, which also included AES and nonexisting potential future funding schemes (e.g., climate premium), as well as international price fluctuations.

All attributes and their levels were selected and refined in consultation with the current literature, previous research in the test site areas, expert opinion, focus group sessions, and the analyses of climate and agro-economic scenarios. Attributes and levels for the CE are summarized in Table 1 and explained in detail below.

The gross margin per ha per year for cash crop cultivation was operationalized based on typical ranges in this area (300, 450, 750, and 1200 €) and an estimation of a potential increase under conditions of climate change, significantly higher than the current gross margins (1650 €). The range of levels for short-rotation forest was determined based on a market research on wood chips revenues across Austria. For grassland, the levels reflect the current range of gross margins in the region, also adjusted to possible effects of climate change.

Environmental premiums per ha per year (AES) differ between the three alternatives. Based on the funding schemes at the time, we increased the available funding and invented a nonexisting climate premium. The premium for cash crop cultivation is mainly paid for catch crops and reflects payments at the time. Currently, Austria does not offer a premium for short-rotation forests. Since wood chips have become increasingly popular as a heating system in many Austrian households in the countryside, a nonexisting climate premium was included to support heating based on renewable energy for this alternative. For grassland, typical payments, which are in most cases around 300 or 600 € per ha and year, were used. Two nonexisting payment levels (900 and 1200 €) were chosen to study opportunities and limits of AES against other land use options.

The duration of cultivation for cash crop remained fixed at 1 year. Climate change is likely to significantly reduce the rotation period of short-rotation forests and make this alternative more attractive in the future. Therefore, the duration for this alternative varied between 15 and 25 years. The duration for grassland is based on the average duration of contracts (7 years in Austria).

The influence of potential price fluctuations was intensively discussed with stakeholders, since international market prices are of increasing importance in this region. As crop-growing decisions are generally made during the spring, only general trends can be considered during this decision-making. Therefore, the alternative-specific levels only include broader statements of expected trends. The price fluctuations are highly significant for cash crop cultivation, relevant for short-rotation forests, and of little importance for grassland. Hence, levels vary between “low” for grassland and “very high” for cash crop cultivation (Table 2).

**Table 2:** Clusters of farmers (based on respondents’ perceived future farm development)

Characteristics	Traditional farm *N* = 14; 9.5 %	Dynamic large farm *N* = 68; 46 %	Farm with perspective *N* = 66; 44.5 %
Agricultural land (average) of which operator owned	63.0 ha	88.7 ha	66.6 ha
	36.9 ha	49.0 ha	37.8 ha
Percentage of part-time farmers	14.5 %	16 %	23 %
Percentage of owners over 50 years of age	35.7 %	10.3 %	16.7 %
Percentage of owners with confirmed successor	40 %	100 %	72 %
Qualification (proportion of college or university degree)	Average	Very high	High
Percentage of organic farms	7.1 %	16.7 %	10.9 %
Percentage who do not perceive climate change as a threat	7.1 %	1.5 %	1.5 %

As floods are possible in this area and as they are likely to increase further under conditions of climate change, a complete crop failure must be taken into consideration. Possible impacts by pondage water differ between the three cultivation alternatives. If the pondage exceeds a few days, short-rotation forests are hardly affected, while cash crop will be significantly damaged. Therefore, the likelihood of complete crop failure for cash crop was selected to occur every “2 years” or “3 years.” In short-rotation forests, a complete crop failure is likely to only occur every 10–25 years, and in areas with grassland every 5, 10 or 15 years.

#### Data Analyses

Data analysis was conducted using IBM SPSS Statistics Version 21 and Microsoft Excel 2011; the choice experiment was analyzed through Latent Gold 5.0. Investigation of the choice model for the whole sample produced reliable results, while a latent class analysis did not yield very insightful outcomes, as class formation generated highly unequally distributed classes with minor significant differences. However, an a priori segmentation by farm type did explain heterogeneity (performed as a ‘known class analysis’ in Latent Gold). The a priori segmentation was based on a principal component analysis with Varimax rotation and a hierarchical cluster analysis applying a Ward’s method clustering of farmers’ perceived future farm development, resulting in three distinct clusters. The computed conditional logit models produced separate, comparable models, in which estimates were compared across classes through Wald(=) statistics. Some estimates, for which the Wald(=) estimates were insignificant, were collapsed across the three segments. The results of the logit regression were then used to design a decision support tool (DST), a predictive tool in Excel. The interface of the DST was designed after the choice experiment, in which all levels of all attributes could be selected individually. The upper section of DST (Table 5, 6 and 7) shows the land use management options, while the lower section gives an insight into the preferences of all farmers (“All farms”) and the three clusters (“Traditional farm,” “Dynamic large farm,” and “Farmer with perspective”). The next section describes all results in detail.

## Results

### Characteristics of Respondents and Farm Structure

The 148 surveyed famers cultivated a total of 11,227 ha of land with an average acreage of 76.3 ha. The majority of the respondents were between 36 and 55 years of age. Almost two-thirds of all participants over the age of 50 had already chosen a successor for their operation. The majority (81 %) farmed full-time, while 19 % were part-time farmers. Most farms were managed conventionally, but 13 % were organically certified. Over 95 % of farmers cultivated cash crops (full-time famers: 76.4 ha average size and 199 ha the largest operation; part-time farmers: 55.9 ha average size and 165 ha the largest operation). Other frequently cultivated crops included wine (29.1 %, average size 6.21 ha), hay meadow (single reaping; 23.0 %, average size 4.7 ha), and ley farming (11.5 %, average size 7.8 ha). Hay meadows with a triple reaping were the least common cultivation method. A comparison with the agro-economic analyses of the region showed that the respondents formed a representative sample for the selected test site (Statistik Austria 2013).

Almost all farmers participated in AES (99.3 %). About two-thirds of the respondents (65.5 %) had already signed conservation-related contracts in the past, and 22 % of them contributed to the conservation of wet meadows in the March–Thaya floodplains. About half of all respondents (48 %) indicated that they would be willing to participate in AES contracts again upon expiry of their current contracts. Another 30 % of all farmers were undecided and 22 % would not sign new contracts. Their main reasons for opposing these contracts were inadequate compensation (10.1 %, *N* = 15), excessive administrative effort (8.8 %, *N* = 15), and lengthy contract periods (8.1 %, *N* = 12).

### Perception of Human-Induced Climate Change by Farmers

Farmers were confronted with the following four statements regarding the occurrence of climate change and were asked to select the one statement closest to their own perception of climate change. Overall, the majority of farmers (64.2 %) already “recognize the first effects of human induced climate change,” while 7.5 % expect to see “significant effects in the near future.” Another 25 % are “undecided if climate change will occur,” and 2 % “do not believe in climate change.” These farmers who do not believe in climate change evaluate the climate change debate as scaremongering and point out that climatic changes go beyond the anthropological records. Nevertheless, when asked about the impact of climate change on agriculture in an open-ended question, 74.3 % of farmers expect effects on agriculture in the province of Lower Austria in the form of “weather extremes,” “more frequent flooding,” “increase of temperatures leading to hotter summers and winters,” “severe droughts,” “increasing fluctuations of temperatures,” “heat waves,” “a decrease in precipitation so that irrigation systems will be necessary,” “uneven distribution of precipitation,” “longer drought periods,” “more and new pests,” “changes in crop rotation,” “altered cultivation potentials,” and “changes in harvest times.”

### Farmers’ Perception of Future Farm Development

**Fig. 4:**
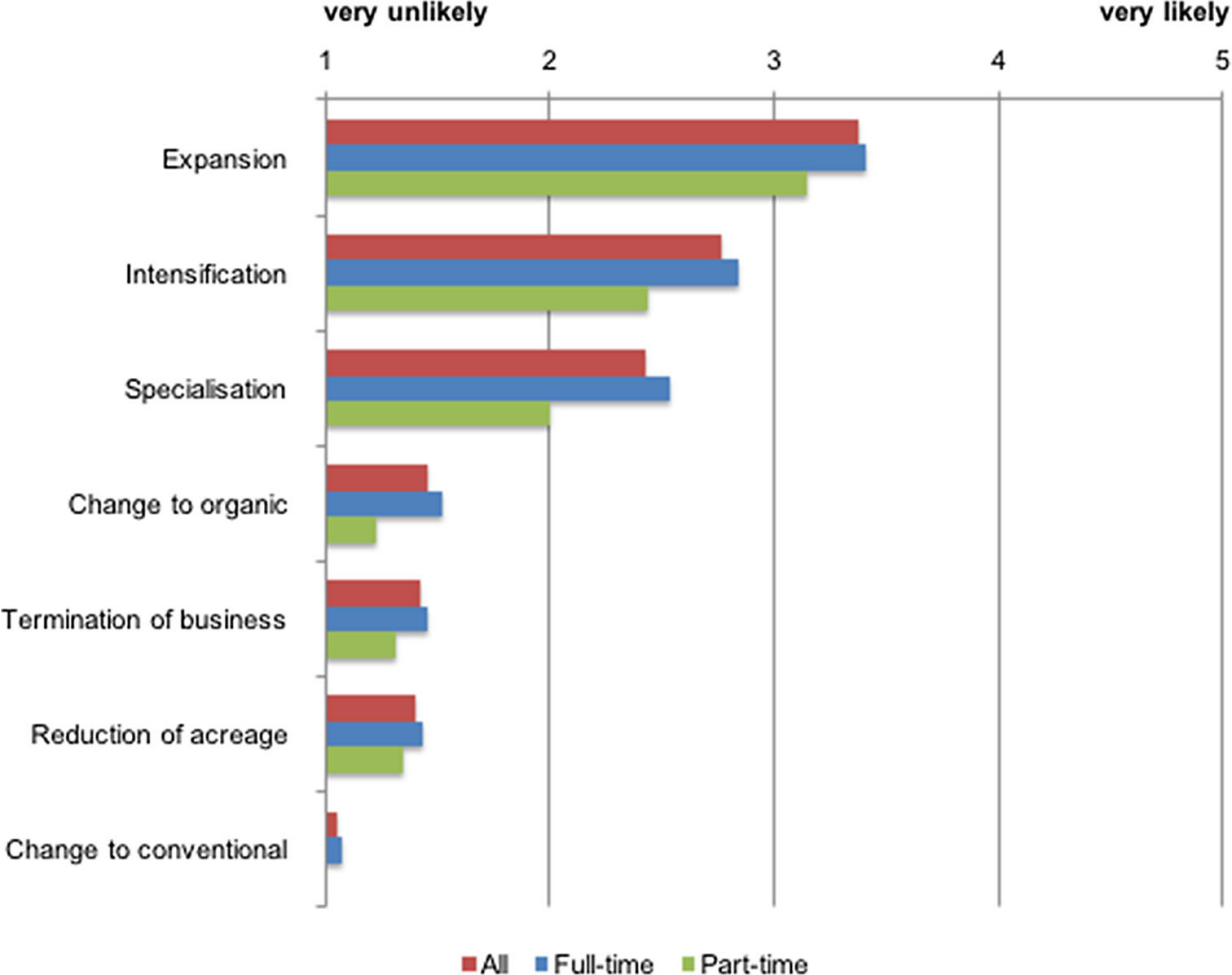
Potential future farm development by farm operating status (mean *N* = 148)

Farmers were asked how they perceived the likely development of their farm in the upcoming 5–10 years on a scale from 1 (very unlikely) to 5 (very likely), (Fig. 4). The majority of respondents planned to expand their farm, intensify farming or specialize in a particular crop; on the other hand, reducing the amount of acreage, changing to a different management model (i.e., conventional vs. organic farming), or terminating the business were the least likely options envisaged. No statistically significant differences emerged between full- and part-time farmers for this question.

Out of this set of seven criteria (Cronbach’s Alpha 0.698) reflecting respondents’ perceived future farm development, the main factors were extracted through a principal component analysis and subsequently used to cluster the sample in a hierarchical cluster analysis. This procedure aimed to investigate if (1) farmers could be meaningfully classified by their future farm development, if (2) these clusters differed in their main characteristics, and if (3) these clusters different in their land use choices (CE).

#### Classification by Future Farm Development

From the principal component analysis, two components emerged with an Eigenvalue greater than one (Kaiser criterion) and factor loadings at an acceptable level above 0.56: the first component combined variables supporting a farm expansion (expansion, intensification, specialization, change to organic), while the other component included indicators of a declining farm operation (termination, reduction, change to conventional). The two components combined explained 65.6 % of variance of the entire sample. A hierarchical cluster analysis applying a Ward’s method to the components resulted in three distinct clusters: Cluster 1—Traditional farms (*N* = 14, 9.5 %), Cluster 2—Dynamic, innovative large farms (*N* = 68, 45.9 %), and Cluster 3—Farms with perspective (*N* = 66, 44.6 %) (Table 2).

#### Main Characteristics of Clusters

The small group “traditional farmers” is the most skeptical regarding climate change. This cluster is significantly older, less educated, and only includes one organically certified operation. Despite being the oldest group, only 40 % have a confirmed successor. The farmers “with perspective” own and operate about the same amount of land, but are significantly younger, better educated, and more interested in organic farming. Almost three quarters already stated a confirmed successor, although the percentage of part-time farmers is significantly higher than in the other two clusters. Farmers in this group are thought of as having perspective, as the majority is highly interested in expanding their operation through purchase or lease of land. A number of these farmers may be in transition to the “dynamic large farm” characterized by significantly larger land holdings. Farmers in the “dynamic large farm” cluster are the most educated, all of them have a confirmed successor, and they state the highest percentage of farms with organic cultivation. All “dynamic farmers” and “farmers with perspective” recognize climate change as a new challenge and already take its influence into consideration.

#### Difference in the Choice Experiment

The three segments differed significantly in their preferences and showed very different preferences and intended behavior for the respective farming alternatives under various climatic conditions and incentive regimes (see following section for details).

### Future Entrepreneurial Decisions of Farmers (Analysis and Results of the CE)

**Fig. 5:**
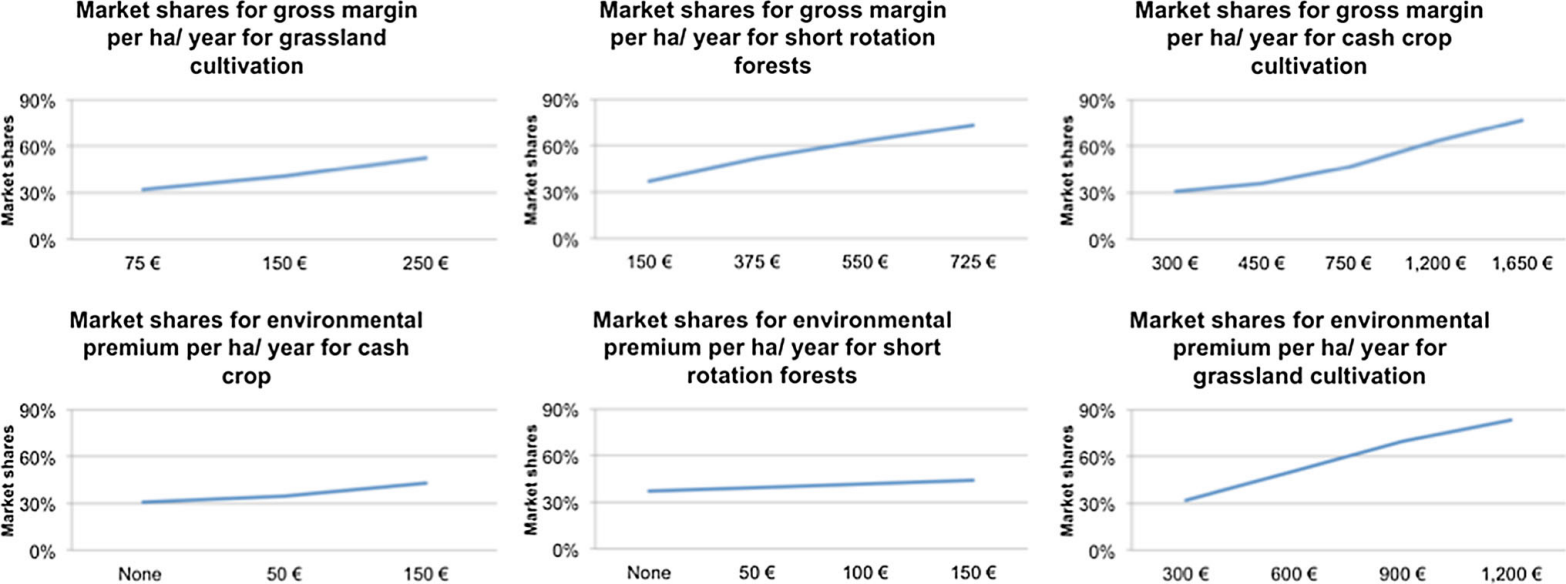
Sensitivity analysis for gross margins and environmental premiums (AES) in terms of likelihood to support scenarios

The results of the conditional logit model for the entire sample (Table 3) show linear estimates for each attribute (separated by cultivation type). All estimates are based on linear coding (nominal coding by level revealed clear linear trends for each attribute). Interactions between the type of cultivation and environmental premium have also been included in the model. The intercepts for the model reveal that, under average baseline conditions, short rotation is much less preferred compared to the two other cultivation methods. The estimates of gross margins and environmental premiums (AES) (Fig. 5) show clear trends and their acceptance increases with higher revenues and support. The preference for AES for grassland cultivation increases further if the premium increases above the current environmental premiums of about 300–600 €. As expected, if the rotation period for short-rotation forest increases to more than 15 years, it is perceived negatively. Possible price fluctuations influence the decision-making in favor of crash crops; much more so than the two other land use options. The likelihood of complete crop failure turned out to be highly significant for cash crop but much less so for short-rotation forests and grassland cultivation. Once the likelihood of a complete crop failure is reduced to every 10 years or more, concerns about failure diminished to an insignificant level.

**Table 3:** One class model

Model for choices					
R² R²(0)	0.157 0.1658				
Attributes	Estimate	s.e.	*z*-value	Wald	*p*-value
Type of management (intercept)					
Cash crop (CC)	0.1129	0.0646	1.7487	27.2058	0.0000
Short rotation (SR)	−0.3009	0.0602	−4.9948		
Grassland (G)	0.1880	0.0565	3.3286		
Gross margin CC	1.5209	0.1753	8.6742	75.2410	0.0000
Gross margin SR	2.7743	0.4174	6.6470	44.1820	0.0000
Gross margin G	0.1749	0.1274	1.3722	1.8831	0.1700
Environmental premium CC	0.3705	0.1338	2.7686	7.6653	0.0056
Environmental premium SR	0.2380	0.1533	1.5523	2.4097	0.1200
Environmental premium G	2.0468	0.2356	8.6891	75.4998	0.0000
Duration SR	−0.1915	0.2119	−0.9038	0.8168	0.3700
Price fluctuation CC	−0.0758	0.0512	−1.4789	2.1872	0.1400
Price fluctuation SR	−0.1633	0.2444	−0.6680	0.4462	0.5000
Failure CC	0.3564	0.0930	3.8335	14.6955	0.0001
Failure SR	−0.0199	0.0830	−0.2399	0.0575	0.8100
Failure G	0.1963	0.1837	1.0690	1.1428	0.2900
Interaction CC gross margin x env. premium	0.0346	0.3649	0.0948	0.0090	0.9200
Interaction SR gross margin x env. premium	0.1283	0.7837	0.1637	0.0268	0.8700
Interaction G gross margin x env. premium	−0.6959	0.3936	−1.7682	3.1266	0.0770

Despite clear trends in the overall model, the three farmer clusters defined above (Table 2) showed very different preferences and intended behavior for the respective farming alternatives under various climatic conditions and incentive regimes. The three segments differed significantly in their preferences for the land use alternatives: Traditional farmers strongly prefer cash crops, and have a serious dislike for grasslands; dynamic large farms prefer grassland; farmers with perspective dislike short-rotation energy crops the most. The alternative-specific model, that also includes interactions, reveals many differences between segments (Table 4), which will become apparent more clearly in the scenarios presented below.

**Table 4:** 3-class (known class) model by farm type

Model for choices													
	Traditional farms			Dynamic large farms			Farms with perspective			Overall			
R²	0.1564			0.2375			0.1157			0.1786			
R²(0)	0.1781			0.2515			0.1246			0.1871			
Attributes	Estimate	s.e.	*z*-value	Estimate	s.e.	*z*-value	Estimate	s.e.	*z*-value	Wald	*p*-value	Wald(=)	*p*-value
Type of management (intercept)													
Cash crop (CC)	0.6883	0.2110	3.2616	0.0838	0.1001	0.8372	0.0230	0.0954	0.2416	44.0316	0.0000	15.8241	0.0033
Short rotation (SR)	−0.1193	0.2142	−0.5569	−0.5569	0.0995	−4.2755	−0.2289	0.0858	−2.6675				
Grassland (G)	−0.5690	0.2379	−2.3918	0.3418	0.0879	3.8907	0.2059	0.0833	2.4721				
Gross margin CC	1.4241	0.5415	2.6301	1.9385	0.2827	6.8579	1.2354	0.2429	5.0852	76.7088	0.0000	3.6702	0.1600
Gross margin SR	2.4395	1.3941	1.7499	3.929	0.7046	5.5764	2.0588	0.5835	3.5284	46.2163	0.0000	4.2836	0.1200
Gross margin G	0.2185	0.1310	1.6685	0.2185	0.1310	1.6685	0.2185	0.1310	1.6685	2.7838	0.0950	0.0000	me
Environmental premium CC	0.4267	0.1377	3.0999	0.4267	0.1377	3.0999	0.4267	0.1377	3.0999	9.6091	0.0019	0.0000	me
Environmental premium SR	0.0171	0.5246	0.0326	0.1309	0.264	0.4960	0.4892	0.2203	2.2204	5.1686	0.1600	1.4375	0.4900
Environmental premium G	0.5276	0.8401	0.6280	2.7464	0.3798	7.2311	1.8649	0.3393	5.4969	82.1839	0.0000	6.9000	0.0320
Duration SR	−0.6953	0.6658	−1.0442	−0.3532	0.3443	−1.0260	0.1125	0.3105	0.3625	2.2796	0.5200	1.7393	0.4200
Price fluctuation CC	−0.0834	0.0526	−1.5878	−0.0834	0.0526	−1.5878	−0.0834	0.0526	−1.5878	2.5210	0.1100	0.0000	me
Price fluctuation SR	−0.1324	0.2498	−0.5298	−0.1324	0.2498	−0.5298	−0.1324	0.2498	−0.5298	0.2807	0.6000	0.0000	me
Failure CC	0.7742	0.3089	2.5062	0.4471	0.1436	3.1139	0.1906	0.1389	1.3721	17.7502	0.0005	3.6914	0.1600
Failure SR	0.2266	0.2731	0.8297	0.0661	0.1332	0.4964	−0.1370	0.1214	−1.1290	2.2165	0.5300	2.1716	0.3400
Failure G	−1.7269	0.7538	−2.2911	0.3764	0.2835	1.3278	0.2981	0.269	1.1082	8.2420	0.0410	7.058	0.0290
Interaction CC gross margin x env. premium	−0.0633	0.3751	−0.1686	−0.0633	0.3751	0.1686	−0.0633	0.3751	−0.1686	0.0284	0.8700	0.0000	me
Interaction SR gross margin x env. premium	−3.3701	2.6562	−1.2688	1.0655	1.3544	0.7867	0.4103	1.0213	0.4017	2.4128	0.4900	2.2674	0.3200
Interaction G gross margin x env. premium	−0.5833	0.4066	−1.4346	−0.5833	0.4066	−1.4346	−0.5833	0.4066	−1.4346	2.0580	0.1500	0.0000	me

*ME* merged effect

These results provide the background for a predictive model or decision support tool (DST), which shows the likely choices by the entire sample and its respective clusters for possible scenarios. Below, we will use the DST to investigate the likely choices of the respective segments with regard to the main research questions on possible new types of agricultural use under conditions of climate change, and the impact of incentives and possible structural policies (AES):

- Scenario 1 analyzes the option to grow short-rotation forests as a new land use option under conditions of climate change.

- Scenario 2 simulates better growing conditions for cash crop, which was supported by a longer growing season and increasing temperatures.

- Scenario 3 analyzes the possible effects by AES and premiums on the decision-making process.

#### Scenario 1: Short-Rotation Forestry: A New Land Use Option Under Conditions of Climate Change

One new land use option, which will become more attractive under conditions of climate change, is the short-rotation forest, which currently requires a rotation period of 20–25 years, even in the excellent growing conditions of these flood plains. The agro-economic analyses showed that both increasing mechanization for this new land use and an increasing price for wood chips might make this form of land use more attractive in the future, especially if the rotation period shortens to 15 years.

Scenario 1 in Table 5 describes the conditions under which most respondents decided in favor of short-rotation forests. We assume a consistent contribution margin for cash crop and short-rotation forestry (around 750 €), while grassland enjoys an environmental premium that is as attractive as the other two options. In that scenario, farmers will take the flood risk of cash crop into account and, therefore, the majority would prefer short-rotation forests. The existing grassland, which is characterized by the same likelihood of complete crop failure (every 10 years) as short-rotation forests, will become significantly less attractive. Especially the traditional farmers will be very attracted to the short-rotation forests (78.62 %) (Table 5).

**Table 5:** Scenario 1—climate change makes short-rotation forestry more attractive/results from DST

Attributes	Land use management alternatives in CE		
	Cash crop cultivation	Short-rotation cultivation	Grassland cultivation
Gross margin per ha per year	€ 750	€ 725	€ 150
Environmental premium per ha per year (AES)	–	–	€ 600
Duration	1 year	15 years	7 years
Potential price fluctuations	Low	Low	Low
Likelihood of complete crop failure (flood)	Every 2 years	Every 10 years	Every 10 years
Preferred alternative (in % of segment)			
All farms	18.35 %	52.64 %	29.01 %
Traditional farm	11.95 %	78.62 %	9.43 %
Dynamic large farm	14.38 %	55.64 %	29.99 %
Farm with perspective	24.14 %	49.64 %	35.21 %

When introducing higher price fluctuations for cash crop, the attractiveness of short-rotation forest would increase further and the share of traditional farmers would increase to 82 % (56.42 % of all farms). If the environmental premium for grassland were to increase to 900 €, the likelihood of growing short-rotation forests would remain high, even though short-rotation forests do not fetch any premiums at the moment. The trends toward heating houses with renewable energy (i.e., wood chips) in rural areas and an already very intensive agricultural land use, could lead to such a premium for short-rotation forests. The sample reacted significantly to this (invented) new premium, especially the “dynamic, large farms” and the “farms with perspective.” The likelihood of a complete cash crop failure every 2 years was another argument in favor of this new land use option. A further effect of climate change can be shown if we assume faster growth of short-rotation forests and a decreased harvest period of 15 years. These changes increase the attractiveness of short-rotation cultivation by another 5–10 %, depending on the segment.

#### Scenario 2: Improved Growing Conditions for Cash Crop

Table 6 describes the effects of a significant increase in the contribution margin of cash crop cultivation, which most agro-economic experts expect to occur due to climate change. To model this scenario, we assume excellent conditions for short-rotation and grassland cultivation, low price fluctuations, the current level of funding, and a moderate likelihood of complete crop failure. Now, the majority of all farmers would shift to cash crop cultivation. Such a shift implies, that even the small, ecologically valuable strips of meadows close to the floodplain would disappear in no time. The typical environmental premium, which is currently about 300 € per ha per year, will not be sufficient to stop this probable trend. Even the small amount of land considered in these CEs (we looked at 3 ha of land per farmer) would be changed under these conditions. Short-rotation cultivation would be chosen by about 22 % of the farmers, grassland cultivation by only about 12 %. Overall, traditional farmers showed the highest likelihood to shift from current grassland cultivation to cash crop.

**Table 6:** Scenario 2—high contribution margin for cash crop will lead to land use shifts

Attributes	Land use management options in CE		
	Cash crop cultivation	Short-rotation cultivation	Grassland cultivation
Gross margin per ha per year	€ 1650	€ 725	€ 250
Environmental premium per ha per year (AES)	–	–	€ 300
Duration	1 year	15 years	7 years
Potential price fluctuations	Low	Low	Low
Likelihood of complete crop failure	Every 3 years	Every 25 years	Every 15 years
Preferred alternative (in % of segment)			
All farmers	64.78 %	22.75 %	12.48 %
Traditional farm	62.13 %	36.52 %	1.35 %
Dynamic large farm	71.13 %	21.59 %	7.28 %
Farm with perspective	64.15 %	17.75 %	18.10 %

#### Scenario 3: Impact of Incentives and Possible Structural Policies (AES)

Since the test site belongs to the European network of protected areas NATURA 2000, EU regulations intend that valuable wet meadows should not be deteriorated. A common mechanism toward this goal is the introduction of contractual measures. In Table 7, we simulated this conservation strategy by offering a very high environmental premium to maintain the extensive use of grassland on the flood plains. To do so, the simulation included an environmental premium of 1200 €, which effectively doubles the current premiums of 300–600 €; the exact amount depends on the specific character of the meadow and the management efforts required. Table 7 shows the farmers’ likely reactions to this high AES for grassland cultivation under the assumption that alternatives do not receive any incentives, that the likelihood of complete crop failure for cash crop is rather high (every 2 years), and that gross margins are high. For short-rotation cultivation in this scenario, we assume a high gross margin under average conditions (duration 20 years, failure every 10 years).

**Table 7:** Scenario 3—significant AES for grassland cultivation have limited effects

Attributes	Land use management options in CE		
	Cash crop cultivation	Short-rotation cultivation	Grassland cultivation
Gross margin per ha per year	€ 1650	€ 725	€ 250
Environmental premium per ha per year (AES)	–	–	€ 1200
Duration	1 year	20 years	7 years
Potential price fluctuations	Low	Low	Low
Likelihood of complete crop failure	Every 2 years	Every 10 years	Every 10 years
Preferred alternative (in % of segment)			
All farms	34.01 %	23.04 %	42.96 %
Traditional farm	38.65 %	47.98 %	13.37 %
Dynamic large farm	26.87 %	14.65 %	58.49 %
Farm with perspective	30.74 %	17.33 %	51.93 %

Table 7 shows that, if all cultivation options provided high contribution margins and a very high AES was implemented to maintain the meadows, 42.96 % of farms would choose this option. A closer look at the three different segments reveals that it is more than half of the “dynamic large farms” (58.49 %) and the “farms with perspective” (51.93 %) that are interested in maintaining the meadows. In other words, even under the condition of extreme incentives, the main goal—to protect these meadows with a very high premium—would not be fully achievable. The traditional farmers were least interested, as only 13 % would continue grassland cultivation in the future. Modifying the risk of potential price fluctuations for cash crop changed the decision slightly in favor of grassland (by about 5 %) for the “dynamic large farm” and “farm with perspective,” but not for the “traditional farm.” Neither did the gross margin of grassland cultivation influence this decision. Only given a low contribution margin for short-rotation cultivation would grassland cultivation become more attractive. If its margin were only around 150 € per ha and year, 75 % of the dynamic large farms and 67 % of the farms with perspective would decide in favor of grassland cultivation. For the traditional farms, this option would remain less attractive (33 %), as the majority would still prefer cash crop cultivation (59 %).

## Discussion and Policy Implications

### Modeling the Effects of Climate Change on Agricultural Land Based on Expert Opinions or Asking Farmers?

Certainly, both approaches are helpful and necessary. However, why one should attempt to involve farmers directly, instead of modeling the likely future developments only, remains a crucial question in climate change research. When Sohl and Claggett (2013) analyzed and discussed the role of land use modeling for decision-making, they discovered that the approach by modelers differs significantly from those by decision makers: modelers are comfortable with abstraction and generalization; they are able to build their models on a limited dataset, apply sophisticated technical analyses, and make modeling decisions in a closed process restricted to technical experts. However, decision makers and public policies focus on practical, transparent, and realistic information. This information should be provided in a flexible and realistic manner, adaptable to specific questions.

Comparing expert-based (Kromp-Kolb et al. 2007) models of climate change impacts on the land use in the test site with the findings of this study, similar overall trends are revealed: a high likelihood of increasing crop margins in the floodplains. The expert model includes specific adaptation measures, such as the potential influence of technical soil management on water consumption, while the possible role of short-rotation forestry is mentioned but not specified. The most crucial aspects, the likelihood of significant land use changes and the loss of remaining grassland, are not covered by the expert-based model at all.

Involving farmers and decision makers directly generated a wealth of insights into their personal values and trade-offs. This involvement also showed how to successfully engage different target groups in adaptive actions, based on certain policy initiatives, such as incentives and funding strategies.

The stated choice survey allowed for a deeper understanding of the individual trade-offs made by famers and of their likely future behavior. Such research findings could help to define policy orientation and, consequently, the social and environmental functions assigned to agriculture (Turner 2007; Bergstrom and Ready 2009; Duke and Johnston 2009; Jeanne and Tina 2012). The findings are especially relevant in the context of the new European policy for agricultural funding and the related AES from 2014 to 2020.

### Individual Farmers’ Perception of Human-Induced Climate Change, Related Opportunities and Risks

The perception of human-induced climate change among farmers in this European region differs greatly from surveys in the US, where farmers frequently denied and disregarded climate change as an important driver of future conditions (Leigh et al. 2013; Prokopy et al. 2013; Mase and Prokopy 2013). Cook and Ma (2014), studying farmers’ perception of climate change in Utah, described a puzzling disconnect: the reported observations about weather and climatic conditions were not in sync with farmers’ belief or disbelief in human-induced climate change. In Austria, descriptions of experts and reported experiences of farmers are rather similar. A large majority of farmers believe in the currently proposed scenarios. They are fully aware of the situation and already invest in irrigation measures to compensate possible impacts. However, compared to respondents in the winter tourism and outdoor recreation industry in Austria (Landauer et al. 2012), the overall perception of human-induced climate change of farmers in Austria is a little lower.

The only exception to farmers’ high awareness of climate change effects in Austria relates to risk behavior. Under conditions of climate change within the immediate range of the March–Thaya floodplains, the flood hazard is likely to increase. Therefore, climate change will lead to new land use options but also to new risks in this area. Farmers’ risk behavior in the choice experiment shows that more risk is taken if the expected contribution margin is very high (for example, see Table 6.). Furthermore, participants with larger farms show a higher risk avoidance behavior than the smaller enterprises and traditional farms. Finally, the overall reaction to economic risks, such as changes of global market prices, is stronger than reactions to climate change phenomena, such as flooding.

### Influence of Incentives and Efficiency of Agro-Economic and Environmental Policy

The choice experiment clearly showed that the decisions by the majority of farmers in the March–Thaya floodplains are driven primarily by the opportunities to earn a higher contribution margin for cash crops. Traditional grassland and short-rotation cultivation, which would constitute a new and attractive land use option due to the extended growing season induced by climatic changes, will therefore continue to represent only a small proportion of overall land use in the future. Even an unrealistic increase of the environmental premium to 1200 € (ÖPUL WF premium) would sway less than half of all farmers to abandon intensive cropping. Interestingly, it was the category of the largest and most modern farms that considered environmental premiums more than the others. On the other hand, risks of the world market and high price fluctuations play a greater role in the decision-making of all farmers than the environmental premium.

### Consequences of the Likely Scenarios to Ecosystem Services and Biodiversity

This study aimed to contribute to a broader understanding of the effects of climate change on multifunctional cultural landscapes, which are currently promoted by European and national agricultural policies. A helpful tool in the discussion of multifunctionality is the concept of ecosystem services; consisting of provisioning, regulating, cultural, and supporting services, the tool has been introduced to identify synergies for biodiversity conservation and other aspects of human welfare improvements (Tallis et al. 2008). In addition to the provisioning function, the study area of the March–Thaya floodplains is also a very important area for outdoor recreation and nature-based tourism (Pröbstl-Haider et al. 2014), and its regulating function, in the context of flood prevention, may become even more relevant in the future.

Climate change is perceived as a major threat to biodiversity in Central Europe. Current research describes an increasing loss of optimal habitats, significant impacts of fragmentation on habitats and the food web, and an increasing relevance of invasive species (Doyle and Ristow 2006; Pauchard et al. 2009; McMullen and Jabbour 2009). Additional, indirect effects of climate change on agriculture can also affect the intensity of land use and its biodiversity. These effects have so far been considered less (Shoyama et al. 2013). A further aggravation is also very likely to impact related ecosystem services. In the case of this study region, a significant loss of meadows must be expected, with significant effects on wild birds (Kelemen-Finan et al. 2011).

The likely development described in the scenarios is that conditions will deteriorate for wild birds, which are already under pressure in the open landscape due to increased fertilization, use of biocides, and increasingly early reaping times (Stübing 2010, Swetnam et al. 2004). The loss of a more diverse landscape and the bird watching opportunities in the March–Thaya floodplains will also affect the cultural services and related regional development options based on tourism. The results show that, based on a behavioral model of famers as decision makers, with the current and even significantly increased environmental premiums, the negative effects on different ecosystem services cannot be mitigated sufficiently to prevent major biodiversity losses.

## Conclusions

Overall, this study showed that, despite current governmental regulations for agricultural matters, climate change will affect traditional landscapes significantly. Wherever possible, farmers will intensify cropping, which will ultimately affect ecosystem services, tourism, and biodiversity. Increasing funding and premiums will only guide future developments of the farming sector if farmers value them as financially adequate (i.e., if they are very high). Current premiums, particularly the environmental premium (ÖPUL), are too low to attract the majority of farmers, and will become even less attractive if climate change permits further yield increases. If premiums are to be adjusted in the future, it is necessary to consider the different decision strategies applied by the various farm segments and operational (farming) types. Without a regionally adapted strategy, the agricultural landscapes are likely to lose their attractive, diverse structure and their suitability for recreation and tourism. These trends may also affect biodiversity and may provoke a discussion about other conservation matters.
